# STOmicsDB: a comprehensive database for spatial transcriptomics data sharing, analysis and visualization

**DOI:** 10.1093/nar/gkad933

**Published:** 2023-11-11

**Authors:** Zhicheng Xu, Weiwen Wang, Tao Yang, Ling Li, Xizheng Ma, Jing Chen, Jieyu Wang, Yan Huang, Joshua Gould, Huifang Lu, Wensi Du, Sunil Kumar Sahu, Fan Yang, Zhiyong Li, Qingjiang Hu, Cong Hua, Shoujie Hu, Yiqun Liu, Jia Cai, Lijin You, Yong Zhang, YuXiang Li, Wenjun Zeng, Ao Chen, Bo Wang, Longqi Liu, Fengzhen Chen, Kailong Ma, Xun Xu, Xiaofeng Wei

**Affiliations:** China National GeneBank, BGI Research, Shenzhen 518120, China; China National GeneBank, BGI Research, Shenzhen 518120, China; China National GeneBank, BGI Research, Shenzhen 518120, China; China National GeneBank, BGI Research, Shenzhen 518120, China; China National GeneBank, BGI Research, Shenzhen 518120, China; China National GeneBank, BGI Research, Shenzhen 518120, China; China National GeneBank, BGI Research, Shenzhen 518120, China; BGI Research, Shenzhen 518083, China; Klarman Cell Observatory, Broad Institute of MIT and Harvard, Cambridge, MA 02142, USA; BGI Research, Shenzhen 518083, China; China National GeneBank, BGI Research, Shenzhen 518120, China; BGI Research, Shenzhen 518083, China; China National GeneBank, BGI Research, Shenzhen 518120, China; BGI Research, Wuhan 430074, China; China National GeneBank, BGI Research, Shenzhen 518120, China; BGI Research, Wuhan 430074, China; China National GeneBank, BGI Research, Shenzhen 518120, China; China National GeneBank, BGI Research, Shenzhen 518120, China; BGI Research, Wuhan 430074, China; China National GeneBank, BGI Research, Shenzhen 518120, China; BGI Research, Shenzhen 518083, China; BGI Research, Shenzhen 518083, China; China National GeneBank, BGI Research, Shenzhen 518120, China; BGI Research, Shenzhen 518083, China; China National GeneBank, BGI Research, Shenzhen 518120, China; BGI Research, Shenzhen 518083, China; BGI Research, Shenzhen 518083, China; China National GeneBank, BGI Research, Shenzhen 518120, China; BGI Research, Shenzhen 518083, China; Guangdong Provincial Key Laboratory of Genome Read and Write, BGI research, Shenzhen 518120, China; China National GeneBank, BGI Research, Shenzhen 518120, China; Guangdong Provincial Genomics Data Center, BGI research, Shenzhen 518120, China

## Abstract

Recent technological developments in spatial transcriptomics allow researchers to measure gene expression of cells and their spatial locations at the single-cell level, generating detailed biological insight into biological processes. A comprehensive database could facilitate the sharing of spatial transcriptomic data and streamline the data acquisition process for researchers. Here, we present the Spatial TranscriptOmics DataBase (STOmicsDB), a database that serves as a one-stop hub for spatial transcriptomics. STOmicsDB integrates 218 manually curated datasets representing 17 species. We annotated cell types, identified spatial regions and genes, and performed cell-cell interaction analysis for these datasets. STOmicsDB features a user-friendly interface for the rapid visualization of millions of cells. To further facilitate the reusability and interoperability of spatial transcriptomic data, we developed standards for spatial transcriptomic data archiving and constructed a spatial transcriptomic data archiving system. Additionally, we offer a distinctive capability of customizing dedicated sub-databases in STOmicsDB for researchers, assisting them in visualizing their spatial transcriptomic analyses. We believe that STOmicsDB could contribute to research insights in the spatial transcriptomics field, including data archiving, sharing, visualization and analysis. STOmicsDB is freely accessible at https://db.cngb.org/stomics/.

## Graphical Abstract

**Graphical Abstract:**
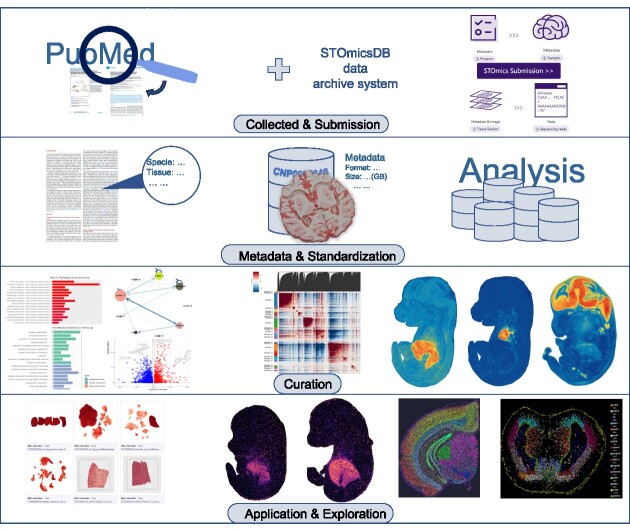

## Introduction

To understand cell development and biological functions, the gene expression profile of cells is a critical element (1,2). Single-cell RNA sequencing (scRNA-seq) technologies characterize gene expression at the single-cell resolution, which is a valuable tool for researchers to elucidate cellular developmental processes. However, scRNA-seq loses spatial information of cells because tissues are dissociated during the experiment (3–6). By contrast, spatial transcriptomic technologies decode the gene expression of cells while retaining spatial information (5,7,8). This huge improvement allows researchers to analyze cell-cell interaction at the single-cell level. With the development of long-reads sequencing (9) and spatial transcriptomic technologies, especially the emergence of high-throughput methods, such as 10× Genomics Visium (https://www.10xgenomics.com/) and the recently released Stereo-seq (10), the studies based on spatial transcriptomic technologies are rapidly accumulating (11). Spatial transcriptomic technologies have been applied to many fields, such as disease research (12–16), organ atlases (4,17–19), organogenesis (10,20,21) and plant biology (22–25). Due to the superiorities of spatial transcriptomic technology in biological research, it was crowned Method of the Year by Nature Methods in 2020 (26).

With the increasing interest in spatial transcriptomics research, there are still challenges remaining. For example, the lack of data archiving standards limits data sharing. The major purpose of a data archiving system is to help other researchers to reuse and re-analyze the data. At present, most spatial transcriptomic data are deposited to Gene Expression Omnibus (GEO) in the National Center for Biotechnology Information (NCBI). However, GEO or other data repositories lack a spatial transcriptomic data archiving standard, resulting in various submission formats. A critical feature of spatial transcriptomics is spatial information. For example, 10x Visium is the most common spatial transcriptomic technology. In general, 10x Visium has three types of information: gene expression data, the spatial information of barcodes and histological images. Most submissions in GEO only include gene expression data but lack spatial information on barcodes or histological images. This absence of spatial information makes the reuse of spatial transcriptomic data challenging. Additionally, cluster marker gene annotation is useful for researchers, but this information is hardly provided in most GEO submissions. Another critical feature of spatial transcriptomics is that the data are obtained from tissue sections. A biological sample may be sliced into different tissue sections. Therefore, biological samples and tissue sections should be recorded during the data archiving. In summary, a specific data archiving standard and the related archiving system for spatial transcriptomics are essential for researchers to reuse and reanalyze spatial transcriptomic data.

For the analysis of spatiotemporal omics data, curation work remains crucial. Curation is a systematic process that involves collecting, organizing, cleaning, validating, standardizing and documenting data, to improve data availability, reliability and comprehensibility. Based on established data standards, integrating data submission systems, and constructing meticulous curation workflows, along with online analysis and visualization modules, are needed to facilitate the discovery of new knowledge through spatiotemporal omics data mining. Several databases focus on spatial transcriptomics, such as SpatialDB (27), SPASCER (28), Aquila (29), SOAR (30) and SODB (31) (Supplementary Table S1). SpatialDB was the first spatial transcriptomics database published in 2019, offering visualization and differential gene expression analysis for 24 datasets (27). SPSCER identified spatially patterned genes/pathways and conducted gene regulatory network analyses and cell-cell interaction analyses for 43 studies (28). Aquila is another database providing multiple analysis and visualization features, including spatial community and spatial co-expression analyses, for approximately 100 datasets (29). Moreover, Aquila distinguishes itself by enabling users to submit their own data for interactive online analysis. SOAR is a database similar to Aqulia, providing spatial variability, adjacency-based cell type interaction and distance-based cell type interaction interaction analysis for 132 datasets (30). SODB stands out by developing an interactive visualization tool, SOView and offering visualization and analysis for more than 100 datasets with SOView (31). While existing spatial transcriptomics databases offer valuable insights, none of them seamlessly integrates data archiving, data analysis and data visualization.

**Figure 1.**
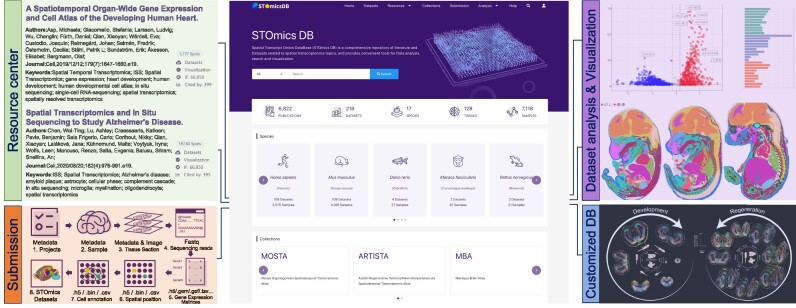
Overview of STOmicsDB. STOmicsDB consists of four modules: the resource center module, the data submission module, the customized database (collections) module and the dataset analysis/visualization module.

Here, we present Spatial TranscriptOmics DataBase (STOmicsDB), a user-friendly database serving as a one-stop service in the spatial transcriptomics field. STOmicsDB has four modules, the resource center module, the data submission module, the customized database (Collection) module and the dataset analysis and visualization module (Figure 1). Firstly, the resource center module integrates 218 manually curated spatial transcriptomic datasets, and more than 6000 spatial multi-omics-related publications for browsing and searching. Subsequently, the data exploration module provides comprehensive visualization and analysis of curated spatial transcriptomic datasets. The customized database module enables collaboration with other researchers to construct specialized spatial transcriptomics databases. Finally, the data submission module provides a spatial transcriptomic data archiving standard and archival system, allowing users to submit and deposit their data to STOmicsDB. In brief, STOmicsDB is a spatial transcriptomics database to analyze and visualize existing datasets and comparative analysis of user data, search-related publications and service of customized database construct and an archiving system of new data. It is anticipated that STOmicsDB could serve as an essential database with a considerable amount of data and functions in the spatial transcriptomics field.

## Materials and methods

### Data collection

To collect spatial multi-omics-related publications, we first searched NCBI PubMed with spatial multi-omics-related terms to obtain candidate articles. Next, we manually curated thousands of candidates to confirm whether they relate to spatial multi-omics. Subsequently, we used those curated candidates as the training set and employed the machine learning method to further select and classify the rest of spatial multi-omics-related publications, resulting in 6822 publications. We used automated scripts to retrieve the information of each publication, which created multidimensional and comprehensive metadata, such as research areas, sample tissue, species, spatial resolutions and publication types.

To collect the spatial transcriptomic dataset candidates, we retrieved the NCBI GEO and European Molecular Biology Laboratory-European Bioinformatics Institute (EMBL-EBI) ArrayExpress resources by searching the term ‘spatial’ and/or spatial transcriptomic technology terms, such as ‘MERFISH’ or ‘10× Visium’. Furthermore, we employed text mining on spatial multi-omics-related publications that were curated before to search for other spatial transcriptomic dataset candidates. Next, we manually curated each dataset candidate to confirm whether it relates to the spatial transcriptome. We also included spatial transcriptomics-associated scRNA-seq datasets (under the same spatial transcriptomic project). In addition, we collected spatial transcriptomic datasets from the 10x genomics website and the SPATIAL research website (https://www.spatialresearch.org/resources-published-datasets). Finally, we curated the datasets which were directly submitted to STOmicsDB. The current version of STOmicsDB contains 218 spatial transcriptomic datasets, covering 128 tissues and 17 species. Each dataset is assigned a unique and permanent accession ID (starting with STDS and following seven-digit numbers, such as STDS0000058). STOmicsDB is a comprehensive spatial transcriptomics database, as it continuously collects new datasets from a wide range of species and tissues. This makes STOmicsDB an essential resource for researchers who want to access and analyze the latest spatial transcriptomics data.

### Data curation and visualization

**Figure 2.**
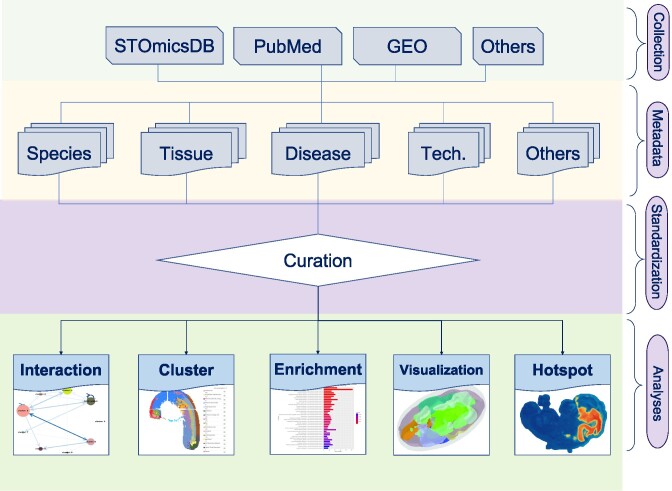
Complete flowchart of dataset curation. This includes data collection, metadata structuring, standardization and data analysis.

We performed multiple analyses to curate the collected datasets and displayed the results (Figure 2). In brief, we first performed cluster annotation using Scanpy (version 1.8.1) (32) with default parameters. For the cluster annotation, we normalized the gene expression data from collected datasets, and then we conducted the principal component analysis (PCA) with the top 2000 highly variable genes to reduce the dimensionality of the data. Next, we calculated the neighborhood graph with PCA results. Uniform Manifold Approximation and Projection (UMAP) analysis and clustering were performed with the Leiden algorithm. Subsequently, we identified cluster markers using the Wilcoxon rank-sum test with ‘scanpy.tl.rank_genes_groups’ function in Scanpy, and genes with an adjusted *P*-value <0.05 were selected. We defined cluster markers with a log_2_ fold change of more than 0.15 as upregulated genes, and performed Gene Ontology (GO) and Kyoto Encyclopedia of Genes and Genomes (KEGG) pathways enrichment analyses on upregulated genes by clusterProfiler (version 4.9.2) (33). For the datasets with spatial location information, we identified spatially specific modules based on highly variable genes with Hotspot (version 1.1.1) (34) under default settings and annotated spatially variable genes using spatialDE (version 1.1.3) (35) with default parameters.

For human and mouse datasets, we annotated cell types for each cluster in datasets using SCINA (version 1.2.0) (36) based on the marker gene database, CellMatch (37). If the datasets contain spatial location information, we also predicted the spatial cell-cell interaction by stLearn (version 0.4.12) (38), according to the spatial context of ligand-receptor pairs in spots.

To visualize the gene expression and spatial location information of curated datasets, we collaborated with the authors of Cirrocumulus (39) and deployed this open-source application on STOmicsDB with customized modifications. Cirrocumulus was designed for rapidly displaying large-scale scRNA-seq data and spatial transcriptomic data. It provides rapid and interactive data visualization. The datasets with Stereo-seq technology also set up the customized visualization system, Stereomap. This ultra-high resolution visualization system is specifically designed for Stereo-seq technology, which can display more than one million cells and accept the ‘gef’ format (a standard format of Stereo-seq technology).

The analyzed results for Cirrocumulus visualization are accessible on the ‘Data’ tab within the individual dataset page. These results are in AnnData format and are named with the ‘*_processed.h5ad’ suffix. The clustering results and cell type annotation results are stored in the .obs attribute of AnnData, with the keys of ‘cluster’ and ‘cell_type’, respectively. The UMPA, PCA and spatial information are stored in the .obsm attribute, with the keys of ‘X_umap’, ‘X_pca’ and ‘spatial’, respectively.

### Data archiving system

**Figure 3.**
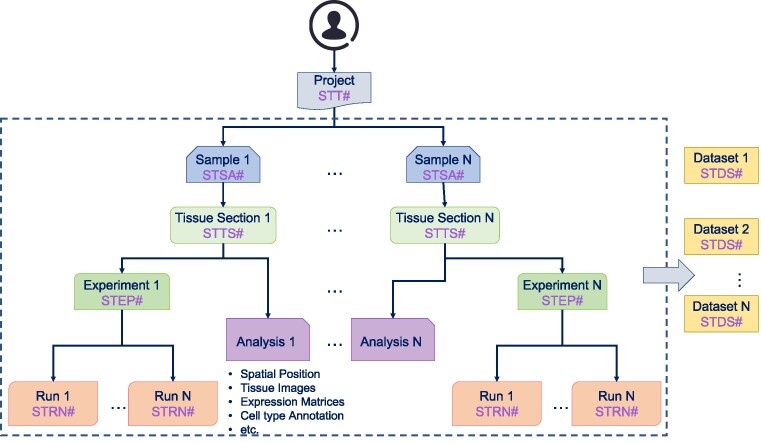
The structure of the spatial transcriptomic archiving system.

We developed standards for spatial transcriptomic data archiving and constructed a spatial transcriptomic data archiving system (Figure 3). To improve the accessibility and reusability of spatial transcriptomic data, our data archiving system requires submitters to provide fully detailed information about their spatial transcriptomic study, including Project, Sample, Tissue section, Experiment & Run and Analysis result (more details can be found in https://stomics-data-archive.readthedocs.io/en/latest/). The project is an overall description of the submitter's study, and each project is assigned a unique accession number for search and dissemination. The sample is the biological source materials used in a study, while the tissue section is the material that is directly sequenced. A sample could be sliced into multiple tissue sections. We require submitters to provide multidimensional metadata to fully describe the tissue sections, such as tissue section size, section thickness, slice position and cryosectioning temperature. For Experiment & Run, we accept fastq/bam format of raw sequencing data along with the relevant metadata. The analysis results include spatial position, gene expression matrices, high-resolution images for tissue section, cell annotation, etc.

Data integrity during each submission and transfer activity is ensured by applying MD5 checksums. All archived data is backed up at a geographically separate data center in case of unexpected events or disasters. Our spatial transcriptomic data archiving system offers two levels of project access privileges, public and controlled. The data from public projects are openly accessible, while access to data from controlled projects is upon request. The access privilege is chosen by the submitter. In addition, we provide submitters with reviewer links for unreleased projects to assist them in the publication process.

### Database construction

We constructed the front-end framework with Vue.js (version 2.6.14) and built the backend using Django (version 2.2) and Python (version 3.7.4). STOmicsDB used PostgreSQL (version 9.6) to store the metadata of publications, and datasets. We used Elasticsearch (version 7.16.2) as the search engine in the resource center of STOmicsDB. We employed MongoDB (version 4.2) and Cirrocumulus to manage and visualize curated datasets. We used Redis (v5.0.4) as the cache to store and manage the data in memory. For task queue management, we applied RabbitMQ (v3.8.13). Nginx (v1.20.1) was used as the reverse proxy server. Currently, STOmicsDB supports the following browsers: Google Chrome (v80.0 and above), Opera (v62.0 and above), Safari (v12.0 and above) and Firefox (v80.0 and above).

## Results

### Overview of STOmicsDB

STOmicsDB consists of four modules: the resource center module, the data submission module, the customized database (Collection) module, and the dataset analysis and visualization module (Figure 1). Users can access each module using the navigation bar on the top of the STOmicsDB home page. The current version of STOmicsDB curates 218 spatial transcriptomic datasets comprising 17 species, covering 25 spatial transcriptomic technologies. STOmicsDB also contains metadata of 6822 spatial multi-omics-related publications. We provide general analyses and visualizations of curated datasets.

### Resource center module

To fulfill the requirement of conveniently accessing resources, STOmicsDB provides a comprehensive spatial resource center for searching and browsing, comprising three sections: (i) Publications; (ii) Samples; (iii) Projects. The spatial multi-omics Publication section contains metadata and concise introductions for each record. Projects encompass an overall description of individual spatial multi-omics research endeavors, often involving multiple samples and datasets. The Samples section provides detailed information on sample handling conditions along with visualization capabilities. Additionally, STOmicsDB incorporates various classifications within each section. For example, the publication section includes research areas, species, tissues, spatial resolutions and publication types, while the Samples section encompasses dataset release dates, species information, tissue details, spatial transcriptomic technologies employed, as well as data quality indicators. Users can specify their area of interest using ontology classifications. Furthermore, every record has its own dedicated page displaying comprehensive information such as summaries and related links.

STOmicsDB offers two user-friendly search methods in the resource center: a quick search and an advanced search. The quick search is accessible through a search box on the homepage, allowing users to select all or specific resources such as publications, datasets, samples and projects using the drop-down list. The advanced search can be easily accessed by clicking the ‘Resources’ button in the top navigation bar. On the ‘Resources’ page, filter conditions are provided in the left sidebar listing section attributes. For instance, on the dataset page, users can refine their selection based on criteria like species, technology, organization time,

### Data submission module

The lack of spatial transcriptomic data archiving standards makes data reuse and re-analysis challenging. We have developed a spatial transcriptomic data archiving system to overcome this obstacle (Figure 3). As of July 2023, STOmicsDB data archiving system has accepted 37 projects, amounting to 16.7TB of data. Among these submissions, 13 projects are public and a total of 85.13TB of public data have been downloaded.

The archiving system serves as a centralized repository for spatial transcriptomic data, allowing researchers to submit their data and associated metadata in a structured manner. This includes the Project, Sample, Tissue section, Experiment & Run and Analysis results (more details can be found at https://stomics-data-archive.readthedocs.io/en/latest/). The Project, Sample and Experiment & Run parts record project information, biological sample information and related experiment information. These three parts are the same as traditional data archiving systems, such as Sequence Read Archive (SRA). Due to the feature of spatial transcriptomics, the data are generated from the tissue section and each sample could have multiple tissue slices. We, therefore, included the tissue section information in our data archiving system. The final part of our submission system is the Analysis result part. Different technologies have different default analysis outputs. For instance, the positions of spatial spots are deposited into a text file by the output of 10x Visium technology, while Stereo-seq stores this information into a binary file. We developed different standards for different technologies according to their features to handle these different features. Additionally, STOmicsDB allows users to submit downstream analysis files, such as marker identification results, differential expression results, or cluster annotation results. Furthermore, the system employs rigorous quality control measures to validate the submitted data and metadata, ensuring accuracy and reliability. This helps to maintain the integrity of the archived spatial transcriptomic datasets and instills confidence in the scientific community. In summary, our spatial transcriptomic data archiving system offers a streamlined submission process and guarantees information integrity through the adoption of a specific metadata/data description format. By facilitating the reusability of spatial transcriptomic data, we aim to accelerate scientific discoveries and promote collaboration in this rapidly evolving field.

### Customized database (collection) module

One significant feature of STOmicsDB is that STOmicsDB provides a customized database service, which we named ‘Collection’. We collaborate with other researchers to construct customized databases that meet their specific needs. Under this collaboration, the researchers provide the data, and we work with them to design the database structure and data visualization. Now, we have constructed six such databases with other researchers: ATRISTA (related to axolotl brain regeneration) (40), MOSTA (related to mouse organogenesis) (10), ZESTA (Zebrafish Embryogenesis Spatiotemporal Transcriptomic Atlas) (21), ACSTA (Arabidopsis Cell-type-specific Spatiotemporal Transcriptomic Atlas) (41), Flysta3D (High-resolution 3D spatiotemporal transcriptomic maps of developing Drosophila embryos and larvae) (42) and MBA (Macaque Brain Atlas) (43). Moreover, we also welcome database hosting. Researchers can construct the spatial transcriptomics database with us and deploy it within STOmicsDB. Users can browse these customized databases by clicking the ‘Collections’ button on the top navigation bar.

### Dataset analysis and visualization module

STOmicsDB offers users the latest spatial transcriptomic datasets as we continue curating public spatial transcriptomic datasets. We integrated the latest curated data along with an online analysis and visualization system. By applying these curations and visualization to spatial transcriptomic datasets, we provide researchers with comprehensive information for numerous spatial transcriptomic studies.

Users can visualize our curated datasets in the ‘Datasets’ section in the navigation bar on the top of the STOmicsDB home page. After selecting the interested dataset, users can find four tabs, ‘Summary’, ‘Visualization’, ‘Data’ and ‘Analysis results’. In the ‘Visualization’ tab, different sections in the same dataset can be chosen through the ‘Sections’ selector on the top. For interactive data visualization, STOmicsDB provides UMAP and spatial map (if the dataset contains spatial information) for curated datasets, which can be switched using the ‘Clustering’ on the sidebar. Users can define each cluster's color and name, and move or zoom the interactive image. If users select one or several genes, the expression heatmap (on UMAP or spatial map) will be displayed on the right and a heatmap, dot plot or violin plot of gene expression will be shown by selecting the ‘HEATMAP’, ‘DOT PLOTS’ or ‘VIOLIN’ button in the top toolbar, respectively. In addition, STOmicsDB shows information on data statistics, cluster marker genes, differential expression analysis based on marker genes, cell-cell interactions, spatially specific modules (Hotspot results) and the spatial marker genes in the ‘Analysis results’ tab. These interactive analyses empower researchers to explore gene expression patterns within their spatial context, identify enriched biological processes and pathways among cluster markers, and investigate intricate cell interactions.

In the ‘Analysis’ section on the navigation bar of the database, there are four online analysis tools for data exploration: ‘SingleR’, ‘Gene search’, ‘Compare’ and ‘Stereomap’. In ‘SingleR’, the SingleR was set up to provide an interactive analysis between user scRNA-seq data and spatial transcriptomic data that STOmicsDB curated. With the help of curated datasets, users can annotate the cell types and obtain spatial information based on their own data. The ‘Gene search’ tool allows users to browse the gene expression pattern among all curated datasets and sections. In the ‘Gene search’ tag, users can select species, tissues and search genes by Ensembl accession ID or gene name. The result displays the related section information from all curated datasets, depending on the given species, tissues and genes. Users can click the section to enter the corresponding visualization interface. The ‘Compare’ tool provides an interactive interface to compare the gene expression pattern or cluster information between two sections or datasets. ‘Stereomap’ is a tool specifically designed for Stereo-seq data. Compared with other visualization tools, Stereomap enables rapid data retrieval and visualization of Stereo-seq, even when dealing with datasets comprising billions of spots.

### User case: candidate genes identification in mice embryo datasets

**Figure 4.**
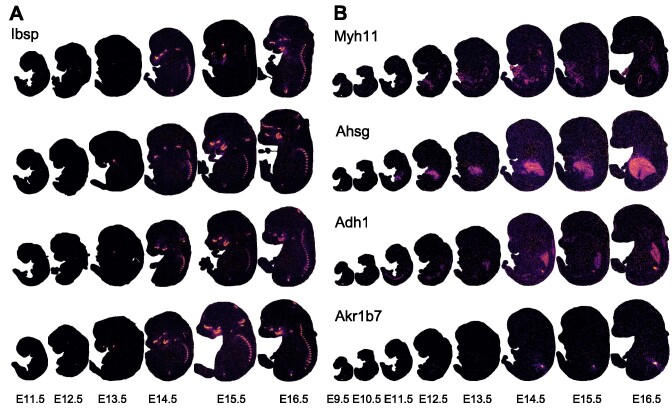
The gene expression pattern of the user case. (**A**) The expression of *Ibsp* during 11.5–16.5 days of mouse embryonic development, with four replicates (row) in each period (column) (details of the section name can be found in Supplementary Table S2). (**B**) The expression of *Myh11*, *Ahsg*, *Adh1* and *Akr1b7* at eight stages (E9.5–E16.5 days) of mouse development.

Based on the curated datasets in STOmicsDB, users can also conduct in-depth data mining using the online analysis and visualization modules. For instance, we selected the curated MOSTA mouse spatial transcriptomic dataset (STDS0000058) to explore the expression patterns of different genes during mouse embryo development. *Ibsp* encodes the protein that acts as the main structural protein of bone matrix, synthesized by skeletal-associated cell types, including hypertrophic chondrocytes, osteoblasts, osteocytes and osteoclasts(44–46). We examined the spatial transcriptomic sections of mouse embryos from 11.5 to 16.5 days, with four replicates for each of the six stages and found that *Ibsp* was expressed in the spine from 14.5 to 16.5 days after mouse development (Figure 4A and Supplementary Table S2). This result indicates that the bone matrix development with the function of *Ibsp* might start from 14.5 days after mouse development. Similarly, we also explored multiple important organs such as the intestine, liver, lung and kidney, revealing the expression patterns of genes such as *Myh11*, *Ahsg*, *Adh1* and *Akr1b7* at different stages of organ development (Figure 4B). Furthermore, using the Hotspot tool, we analyzed and validated other locally spatially specific gene expression patterns during mouse development, finding more evidence of genes expressed in different stages of organ development.

## Discussion

STOmicsDB is a comprehensive spatial transcriptomics database that makes efforts to the connectivity, reusability and interoperability of spatial transcriptomic resources. STOmicsDB provides multi-dimensional analysis results, such as differential gene expression analysis, spatially patterned regions/genes analysis, and also incorporates relevant literature to enhance researchers' understanding and utilization of the provided information. The database aims to facilitate the comprehension and application of spatial transcriptomics within the research community by providing an accessible and all-encompassing data resource. Moreover, we have developed a spatial transcriptomic data archiving system to facilitate the reusability of spatial transcriptomic data. This system streamlines the spatial transcriptomic data submission process and ensures information integrity by adopting a specific metadata/data description format for spatial transcriptomic data. The purpose of our system is to simplify and standardize the process of archiving spatial transcriptomic data, making it easier for researchers to share and access this valuable resource. By implementing the specific metadata/data standard, we ensure that the information accompanying the spatial transcriptomic data is consistent and comprehensive, enabling other researchers to understand and utilize the dataset effectively. Additionally, interactive visualization tools and downstream analysis features have been integrated into STOmicsDB, offering users an intuitive and efficient way to comprehend and analyze the curated datasets. Overall, STOmicsDB is a comprehensive resource for spatial transcriptomics research, and it is expected to greatly benefit the spatial transcriptomic community.

In the future, we will improve STOmicsDB in the following directions. First, we will continue to curate datasets and publications as new spatial transcriptomics projects are published. Next, we plan to create multi-level interactions among publications and datasets. For example, these multi-level interactions will show which tool is most used in papers, or how many spatial multi-omics relevant papers a specific author has published. This will make it easier for users to easily obtain the latest and most comprehensive information on spatial transcriptomics. Furthermore, we will develop a comprehensive online spatial transcriptomics submission system. Although we have collected and curated 25 spatial transcriptomic technologies, we still struggle to support interactive visualization for every technology. Finally, we plan to integrate the marker genes of curated datasets of the same species or the same organ to generate gene networks or relevant atlases. These networks may improve our ability to efficiently characterize biological insight into cells and tissues.

## Supplementary Material

gkad933_Supplemental_File

## Data Availability

STOmicsDB is freely accessible at https://db.cngb.org/stomics/. The data curation results described in this manuscript are freely available to access and can be downloaded at https://db.cngb.org/stomics/.
