# Supplementary material for: STOmicsDB: a comprehensive database for spatial transcriptomics data sharing, analysis and visualization
Source: Nucleic Acids Res. 2023 Nov 11;52(D1):D1053–61. doi: 10.1093/nar/gkad933 (PMC10767841; doi:10.1093/nar/gkad933)
Supplement: gkad933_Supplemental_File [file gkad933_supplemental_file.pdf]

Supplementary Table 1. The comparison of features among different spatial transcriptome databases (up to September 20th, 2023). The information of STOmicsDB is bolded.

Database	Species	Technologies	Datasets	Samples	Literatures	Data visualization	Cell type annotation	Spatial pattern gene	Cell-cell interaction	Analysis of user's data	Data archiving system
**SODB**	11	26	151	2,755	no	yes	yes	no	no	no	no
**Aquila**	5	16	110	6,500	yes	yes	yes	yes	yes	yes	no
**SPASCER**	4	8	43	1,082	no	yes	yes	yes	yes	no	no
**SOAR**	6	9	145	1,735	no	yes	yes	yes	yes	no	no
**SpatialDB**	5	8	24	305	no	yes	no	no	no	no	no
**STOmicsDB**	**17**	**25**	**218**	**7,118**	**yes**	**yes**	**yes**	**yes**	**yes**	**yes**	**yes**

Supplementary Table 2. The corresponding section names in the Figure 4.

	Column1	Column2	Column3	Column4	Column5	Column6
**Row1**	E11.5_E1S1	E12.5_E1S2	E13.5_E1S1	E14.5_E1S1	E15.5_E1S2	E16.5_E2S9
**Row2**	E11.5_E1S2	E12.5_E1S4	E13.5_E1S2	E14.5_E1S2	E15.5_E1S3	E16.5_E2S4
**Row3**	E11.5_E1S3	E12.5_E1S5	E13.5_E1S3	E14.5_E2S1	E15.5_E1S4	E16.5_E1S4
**Row4**	E11.5_E1S4	E12.5_E2S1	E13.5_E1S4	E14.5_E2S2	E15.5_E2S1	E16.5_E1S1

Note: The first 'E' indicates the day of the development, the second 'E' indicates the mouse number, and the 'S' indicates the section number. eg. The E11.5_E1S1 means the 11.5th day, No.1 mouse, the No.1 section.
